# Random access to palatable food stimulates similar addiction-like responses as a fixed schedule, but only a fixed schedule elicits anticipatory activation

**DOI:** 10.1038/s41598-019-54540-0

**Published:** 2019-12-03

**Authors:** Geovanni Muñoz-Escobar, Natalí N. Guerrero-Vargas, Carolina Escobar

**Affiliations:** Departamento de Anatomía, Facultad de Medicina, Universidad Nacional Autónoma de México, Ciudad de México, Mexico

**Keywords:** Motivation, Reward

## Abstract

Restricted intermittent food access to palatable food (PF) induces addiction-like behaviors and plastic changes in corticolimbic brain areas. Intermittent access protocols normally schedule PF to a fixed time, enabling animals to predict the arrival of PF. Because outside the laboratory the presence of PF may occur in a random unpredictable manner, the present study explored whether random access to PF would stimulate similar addiction-like responses as observed under a fixed scheduled. Rats were randomly assigned to a control group without chocolate access, to *ad libitum* access to chocolate, to fixed intermittent access (CH-F), or to random unpredictable access (CH-R) to chocolate. Only the CH-F group developed behavioral and core temperature anticipation to PF access. Both groups exposed to intermittent access to PF showed binge eating, increased effort behaviors to obtain chocolate, as well as high FosB/ΔFosB in corticolimbic areas. Moreover, FosB/ΔFosB in all areas correlated with the intensity of binge eating and effort behaviors. We conclude that both conditions of intermittent access to PF stimulate addiction-like behaviors and FosB/ΔFosB accumulation in brain reward areas; while only a fixed schedule, which provides a time clue, elicited anticipatory activation, which is strongly associated with craving behaviors and may favor relapse during withdrawal.

## Introduction

High caloric palatable food stimulates overconsumption, which then can lead to overweight and obesity. Palatable food (PF) normally contains high amounts of sugar, fat or salt, the combination of these flavors exerts an hedonic effect that stimulates food intake in the absence of hunger, which is driven by the reward properties and is termed non-homeostatic feeding^1,2^.

Restricted or intermittent access to PF triggers an increased food driven response suggested to have similar properties as addiction-like behaviors observed for drugs^3–6^. In rodents the food addiction-like behavior includes craving, binge eating, effort behaviors to obtain food and withdrawal symptoms when the expected food is not provided^3^. Contrasting, free access to PF leads to overweight and/or obesity; however, does not trigger addiction-like behavior^7^.

Restricted access to PF elicits craving, characterized by increased behavioral activation associated with seeking and expectation. It occurs in anticipation to the expected access to PF or after a period of deprivation. It is suggested that the intensity of this anticipatory activation is correlated with the motivation for consuming the specific restricted food^8,9^. Anticipatory activity has been extensively described in rodents exposed to restricted feeding schedules either to normal chow or to PF^10^. This behavioral activation is accompanied by physiological changes that include an anticipatory rise of circulating corticosterone levels^11^, a rise in temperature^12^ and production of digestive enzymes and hormones^13^ among others.

Restricted access to PF also induces binge-like eating events^1^. Binge-like behavior is defined as overconsumption of food in a brief period of time, as compared to the normal food consumption under similar conditions in a similar period of time^14^. In rodents, overconsumption occurs when animals consume 20–50% or more of the expected intake for the same interval. Rats fed daily intermittent PF increase progressively their food intake, especially during the first hour^15,16^ which is called escalation and resembles a tolerance process^17^. In contrast, rats offered PF *ad libitum* consume similar amounts distributed throughout 24 h.

Restricted access to PF also elicits effort behaviors in order to obtain the food or drugs. Effort behavior is usually evaluated with an operant task exploring the progressive ratio and the amount of lever pressing that the rodent is willing to display for a reward^18,19^. Effort behavior is also observed in rodents challenged to cross an electrified grid or to endure other aversive stimuli in order to obtain the reward^20^. We developed a test using a wire-mesh box that contains a piece of chocolate and measured the effort behavior of the rat trying to extract the PF^16^. This test does not require training and does not expose rodents to an aversive stimulus. In a previous study we described that rats exposed to restricted access to PF exhibit strong interaction with a wire-mesh box containing chocolate^16^.

Food reward responses are driven by a brain network that includes the ventral tegmental area in the midbrain, the nucleus accumbens, the hippocampus, prefrontal cortex (PFC) and basolateral amygdala^21^. These same areas exhibit high c-Fos activation in animals expecting scheduled access to chocolate including the paraventricular nucleus in the thalamus (PVT)^16,22^, which is suggested to play a relevant role for anticipation due to the integration of orexigenic and dopaminergic projection with behavior^23^. It is suggested that the interaction between areas in this network is critical for inducing craving, anticipation and reward seeking behavior^24,25^. Also, high c-Fos activation was described in the dorsomedial hypothalamus (DMH), and the arcuate nucleus (ARC) associated with anticipatory activation and in response to food ingestion^26,27^. In corticolimbic areas the accumulation of the protein FosB/ΔFosB in these areas is reported after drug administration and after drug interruption^28^. The protein FosB/ΔFosB is a transcription factor involved in triggering plastic adaptations in neurons of the reward system^29,30^. Specifically it is suggested that FosB/ΔFosB stimulates dendritic growth, favoring the enforcement of synaptic contacts that will induce and maintain the addiction-like response, therefore the accumulation of FosB/ΔFosB is suggested to be a biomarker of addiction^31^.

In a previous study^32^ we compared rats exposed to a daily fixed restricted feeding schedule with rats exposed to an unpredictable restricted feeding schedule. We reported that rats under an unpredictable schedule were not able to binge as much as the rats exposed to the fixed schedule, they were unable to develop anticipatory activation and did not exhibit metabolic adaptations necessary for the scheduled mealtime. Studies aimed to observe addiction-like behaviors to PF preferentially use an intermittent access protocol, where the food is scheduled to a fixed time of the day or night^33–35^. Because outside the laboratory the presence of a PF may occur in a random unpredictable manner, the aim of the present study was to explore whether the fixed schedule is necessary and contributes to the development of the addiction-like response to PF. We hypothesized that the intermittent access in a random unpredictable manner would elicit diminished addiction-like behaviors and diminished accumulation of FosB/ΔFosB, while the incorporation of a temporal cue with a fixed schedule would potentiate the development of addiction-like behaviors. In rats exposed to *ad libitum*, fixed or random intermittent access to chocolate we compared effort behavior, binge eating, and anticipatory activation, as well as FosB/ΔFosB expression in corticolimbic brain areas, and hypothalamic areas, as an indicator of neuronal plastic changes.

## Results

### Scheduled restricted access to palatable food is necessary for anticipatory activation

**Figure 1:**
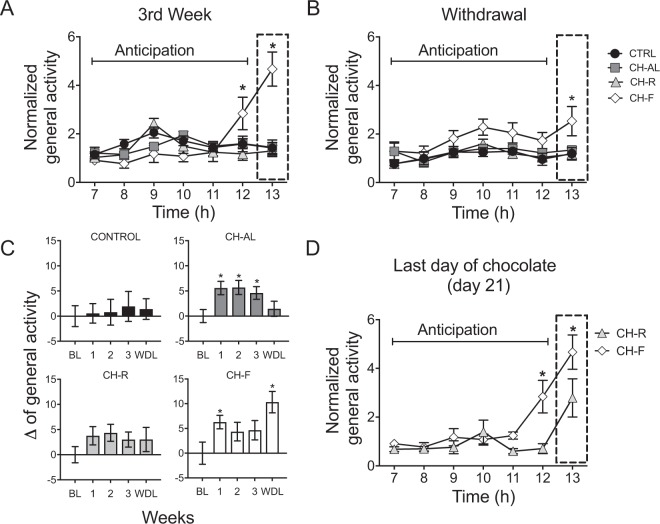
Scheduled restricted access to chocolate induces anticipatory activation. (**A**) Mean general activity for control (CTRL), chocolate ad-libitum (CH-AL), chocolate random (CH-R) and chocolate fixed (CH-F) rats, corresponding to the first 6 h preceding chocolate access and for 1 h during chocolate intake. The CH-F group showed an increase in general activity 1 h before chocolate access (at 12 h), and strong activation during chocolate intake (13 h, dotted rectangle) vs all groups (*P < 0.05 Tukey post hoc). (**B**) Mean general activity for the same 6 h interval during the withdrawal week. CH-F rats showed a persistent increase of activation at 13 h, the time when chocolate was ingested (dotted rectangle); this was significantly different from all groups. (**C**) Difference (Δ) from baseline (BL) in general activity for the 12 h of light phase along the 3 weeks of chocolate access and 1 week of withdrawal (WDL). CH-AL and CH-F groups showed a significant increase as compared to BL. (**D**) Chocolate driven anticipatory activation in the last day of chocolate exposure (day 21) for both restricted access groups (CH-R and CH-F). Data are expressed as the mean ± SEM (n = 8/group). Asterisk indicates difference from other groups (P > 0.05) and + indicates significant difference from other times points in the same group (P > 0.05). For C, the *indicates statistical difference from BL (P > 0.05).

During the baseline all groups showed a clear daily pattern of general activity, characterized by low activity counts during the light phase (18–20% of daily total activity) and high values in the dark phase (80% of daily total activity). During the experimental weeks, control (CTRL), chocolate *ad-libitum* (CH-AL), chocolate random (CH-R) and chocolate fixed (CH-F) groups maintained a day-night activity pattern (Supplementary Fig. 1). Only the CH-F group developed anticipatory activation, characterized by a significant increase of activity counts 1 h preceding chocolate access (Fig. 1A). Contrasting, CH-R and CH-AL rats did not develop anticipation and their activity values remained similar along the first 6 h in the light phase (Fig. 1A). The two-way ANOVA for RM indicated significant interaction for groups/hours (F_(18,168)_ = 9.62; P < 0.0001). During the withdrawal week (WDL) the CH-F group exhibited persistent activation at the expected chocolate time (Fig. 1B). The two-way ANOVA for RM indicated a significant effect for groups (F_(6,168)_ = 4.85; P < 0.0001) and hours (F_(3,28)_ = 5.16; P < 0.0057).

To better observe the impact of chocolate intake, the change of general activity (Δ) in the light phase along the weeks was compared with the baseline activity (Fig. 1C). While the CTRL group maintained similar day-night activity along the protocol, CH-AL and CH-F groups significantly increased their activation in the light phase (Fig. 1C). The one-way ANOVA for RM indicated significant difference along the weeks for the CH-AL (F_(4,35)_ = 6.936; P < 0,0003) and for the CH-F (F_(4,35)_ = 4.96; P < 0,0028) and no significant effects for CH-R and CTRL groups. The two-way ANOVA for RM indicated differences between groups (F_(4,140)_ = 3.317; P = 0.0125) and time (F_(3,140)_ = 4.103; P = 0.0080). The poshoc intergroup comparison indicated that during the WDL week the CH-F group exhibited higher activation at the expected chocolate time as compared to all groups. On day 20 the CH-R rats received the piece of chocolate at 13 h; however, on day 21 they did not show anticipation to chocolate as observed in the CH-F group (Fig. 1D), indicating that regularity and temporality to chocolate access is necessary to develop anticipatory activation. The two-way ANOVA for RM indicated significant difference between groups (F_(7,91)_ = 3.086; P < 0.005).

**Figure 2:**
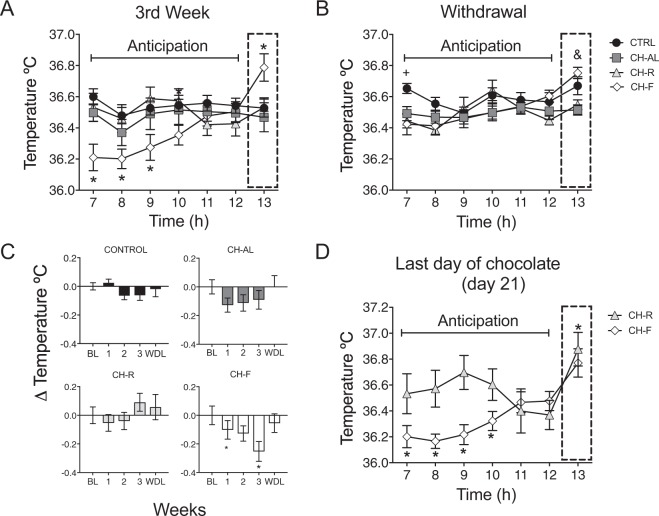
Scheduled restricted access to chocolate induces an anticipatory decrease followed by a progressive increase in core body temperature. (**A**) Mean core body temperature during 6 h before the access to chocolate. Control (CTRL), chocolate ad-libitum (CH-AL), chocolate random (CH-R) and chocolate fixed (CH-F) rats. On week 3, CH-F group showed a decrease in temperature 6 h preceding scheduled chocolate access followed by a progressive increase starting 4 h before the scheduled chocolate access. This was not observed in the other groups. The dotted rectangle indicates the moment of daily chocolate access for CH-F group. (**B**) Mean of daily core temperature during the withdrawal (WDL) period for the same 6 h interval, the dotted rectangle indicates the expected moment of chocolate access. (**C**) Mean temperature changes in core temperature during the protocol of chocolate access as compared with the BL. (**D**) Last day (day 21) of chocolate exposure after both restricted access groups had received chocolate at 13 h (dotted rectangle). Data are expressed as the mean ± SEM (n = 8/group). The two-way ANOVA for RM indicated a significant difference among groups (P < 0.05). For A and B, the Tuckey’s test indicated statistical difference * from all groups vs CTRL and & from CH-AL vs CTRL (P > 0.05). For C, the Tuckey’s test indicated a statistical difference * from base line (P > 0.05). For D, the Tuckey’s test indicated statistical difference * from CH-R (P > 0.05).

All groups showed clear daily rhythms of core temperature during BL and the experimental weeks (Supplementary Fig. 2). In the 3rd week of chocolate exposure, the CH-F group exhibited an anticipatory temperature change characterized by a significant decrease starting 6 h preceding chocolate access followed by a progressive increase until chocolate time (Fig. 2A). This increase started 4 h before chocolate time. The two-way ANOVA for RM, indicated significant interaction between groups and time (F_(18,162)_ = 7.73; P < 0.0001). A similar analysis was performed during the WDL week and for the CH-F group no persistence of the anticipatory decrement of temperature was observed, however the increase associated with chocolate access was still present (Fig. 2B). The two-way ANOVA for RM indicated significant effects for the interaction groups/h (F_(18,126)_ = 2.77; P = 0.0005).

To better analyze the anticipatory decrement in temperature associated with chocolate intake, the mean temperature in the light phase along the 3 weeks of chocolate access and WDL was compared with the BL (Δ; Fig. 2C). The one-way ANOVA for RM indicated significant changes along the weeks for the CH-F group (F_(3,36)_ = 6.385; P < 0.007) and no significant changes for CTRL, CH-AL and CH-R groups. The two-way ANOVA for RM indicated differences in time (F_(3,140)_ = 5.383; P = 0.0016) and not between groups. The poshoc intergroup comparison indicated that during the 3rd week the CH-F group exhibited higher activation at the expected chocolate time as compared to all.

On day 21 after one day with the chocolate scheduled at 13 h, the CH-R group did not develop the anticipatory decrease in temperature; however, it exhibited a peak at the moment of the expected chocolate access, similar to the CH-F group (Fig. 2D). The two-way ANOVA for RM indicated a statistical difference between CH-F and CH-R (F_(7,49)_ = 6.89; P < 0.0001).

### Restricted access to chocolate induces binge eating behavior

**Figure 3:**
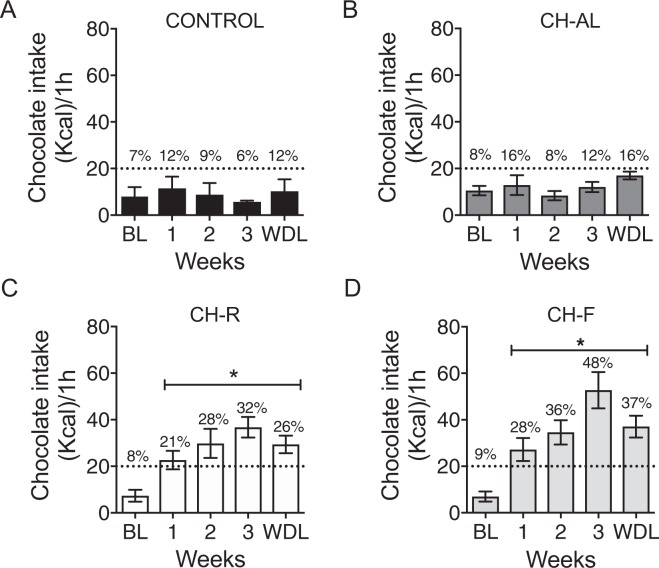
Restricted access to chocolate induces binge eating behavior. Mean calories of chocolate intake during 1 h at the end of each experimental week and after 1 week of withdrawal (WDL). (**A**) Control rats (CTRL), without chocolate, (**B**) Chocolate ad libitum rats (CH-AL), (**C**) Chocolate random access (CH-R) and (**D**) Chocolate fixed (CH-F) groups. In the binge test, CH-restricted access groups showed chocolate overconsumption of more than 20% of their daily caloric intake (dotted horizontal lines). The percentages indicate the proportion for the total mean daily caloric consumption. Data are expressed as the mean ± SEM (n = 8/group). The asterisk indicates a statistical difference from base line (P > 0.05).

For the Binge test, one set of rats received on Fridays a complete bar of chocolate from 13 h to 14 h. For the CTRL group the caloric intake of chocolate for 1 h did not differ along the weeks of the protocol (Fig. 3A) and it did not reach the binge criteria of 20% of the daily caloric intake. The one-way ANOVA for RM indicated no significant difference along the weeks (F_(4,35)_ = 0.412; P = 0.68; Fig. 3A). For the CH-AL rats the caloric ingestion of chocolate during 1 h also remained below the 20% along the weeks and did not show significant difference along the weeks (F_(4,35)_ = 1.426; P = 0.27; Fig. 3B). In contrast, both chocolate restricted access groups, CH-R and CH-F surpassed the criteria of 20% of caloric intake, which coincides with the criteria for binge eating as early as week 1. The chocolate intake increased over the weeks, reaching on week 3, 32% and 42% of daily kilocalorie intake for CH-R and CH-F respectively (Fig. 3C,D). An intergroup comparison indicated no differences between groups (F_(4,56)_ = 0.8408; P = 0.50).

After the WDL week, the caloric intake for the CH-R and CH-F groups in 1 h remained high, reaching a 26% and 37% of their total daily kcal intake respectively. The one-way ANOVA for RM indicated significant difference along the weeks for CH-R (F_(2,15)_ = 7.09; P < 0.005) and CH-F (F_(1,13_ = 10.83; P = 0.0015).

Independent of the binge test, the daily chocolate and chow intake was recorded. The CH-AL group rats increased their daily chocolate intake along the weeks and reduced their regular food intake (Supplementary Fig. 3C), more over the CH-R and CH-F rats adjusted their daily chow intake in order to maintain constant values in their total caloric intake in spite of the 5 g of chocolate consumed daily (Supplementary Fig. 3D,E). The daily chocolate intake impacted on body weight gain of both chocolate restricted groups (CH-R and CH-F), while chocolate *ad libitum* did not produce a significant overweight as compared to CTRL (Supplementary Fig. 3A). The two-way ANOVA for RM indicated significant interaction for groups/weeks (F_(12,112)_ = 3.628; P = 0.0001).

### The wire-mesh box test: effort for chocolate

**Figure 4:**
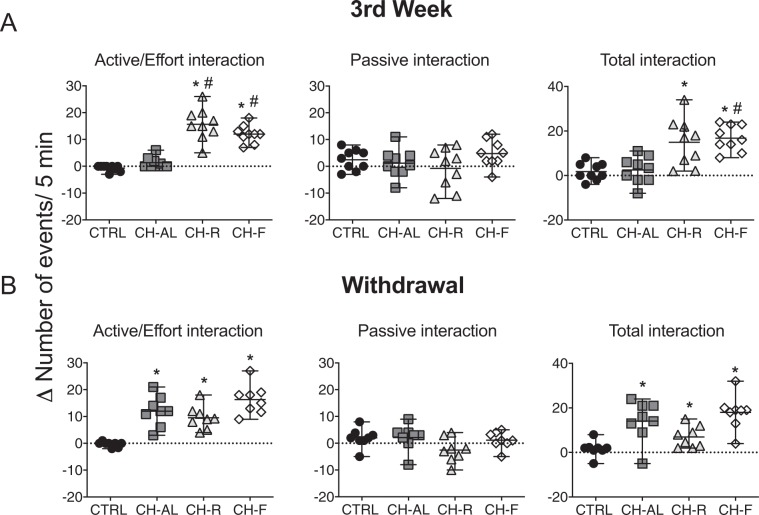
Restricted access to chocolate induces effort behaviors in order to obtain the chocolate. Difference (Δ) between the number of events displayed to the empty box vs. the box containing 5 g of chocolate for Control (CTRL), chocolate ad-libitum (CH-AL), chocolate random (CH-R) and chocolate fixed (CH-F) rats. (**A,D**) Active/Effort; (**B,C**) Passive interaction and. (**C,F**) Total interaction at the end of week 3 of chocolate access (top) and after 1 week of withdrawal (bottom). Data are expressed as the median ± range (n = 8/group). The Dunn’s test indicated significant difference * from CTRL group and # a significant difference from CH-AL rats (P < 0.05).

In the wire-mesh box test CTRL and CH-AL groups showed low effort events directed to extract the chocolate. In contrast the CH-R and CH-F groups showed high effort behaviors aimed at extracting the chocolate (Fig. 4A). The Kruskal Wallis analysis indicated significant difference between the groups (H(4) = 28.80 P < 0.0001). All groups showed similar passive interaction with the box as indicated by sniffing and approaching the box (Fig. 4A) and the Kruskal Wallis test indicated no significant difference among groups (H(4) = 3.042 P < 0.38). Rats with restricted access to chocolate (CH-R and CH-F) exhibited more total interaction than CTRL group. The Kruskal Wallis analysis indicated significant difference between the groups (H(4) = 19.27 P < 0.0002; Fig. 4A).

After one week of WDL, all groups exposed to chocolate, including the CH-AL exhibited increased effort behaviors to obtain the chocolate and exhibited more total interaction with the box as compared to the CTRL group (Fig. 4B). The Kruskal Wallis analysis indicated significant difference between the groups for the effort behaviors (H(4) = 20.87 P < 0.0001); Fig. 4B) and for the total interaction (H(4) = 17.07 P < 0.0007.; Fig. 4F); while no significant difference was observed for the passive interaction (H(4) = 5.037 P = 0.16; Fig. 4B).

### Restricted access to chocolate increased FosB/ΔFosB positive cells in corticolimbic areas

**Figure 5:**
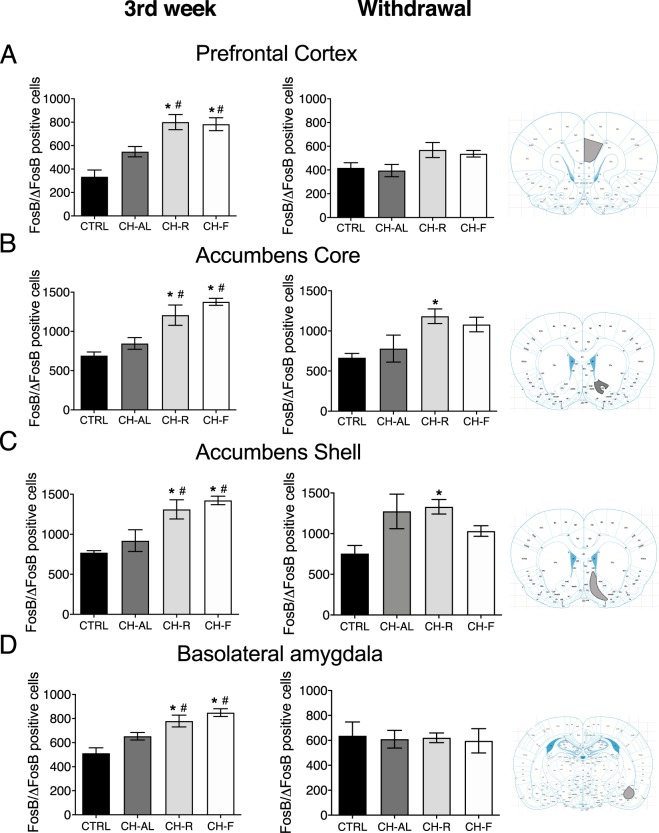
Restricted access to chocolate induces enhanced FosB/ΔFosB-expression in corticolimbic areas. Number of FoB/ΔFosB positive cells at the end of the 3^rd^ week of chocolate exposure (left column) and at the end of the withdrawal period (right column) for Control (CTRL), chocolate ad-libitum (CH-AL), chocolate random (CH-R) and chocolate fixed (CH-F) rats. (**A**) Prefrontal cortex, (**B**) Accumbens Core, (**C**) Accumbens Shell and (**D**) Basolateral amygdala. Data are expressed as mean ± SEM (n = 4–8/group). The Tuckey’s test indicated statistical difference from CTRL indicated with * and statistical difference from CH-AL indicated with ^#^(P < 0.05).

After 21 days of chocolate access, more FosB/ΔFosB positive cells were counted in the corticolimbic areas for the CH-R and CH-F groups as compared to CTRL and CH-AL rats (Fig. 5A–D left column). The one-way ANOVA indicated significant difference between groups in the PFC (F_(3,28)_ = 15.70; P < 0.0001), NAccCore (F_(3,22)_ = 11.60; P < 0.0001), NAccShell F_(3,27)_ = 17.23; P < 0.0001) and BLA (F_(3,27)_ = 11.18; P < 0.0001). The number of FosB/ΔFosB positive cells was also evaluated in hypothalamic nuclei dorsomedial hypothalamus (DMH) and arcuate nucleus (ARC), and the gating nuclei paraventricular thalamus (PVT). However, no differences in FosB/ΔFosB positive cells were observed between groups in the PVT (F_(3,13)_ = 0.44; P = 0.7245; Supplementary Fig. 4A), ARC (F_(3,13)_ = 0.89; P = 0.4688; Supplementary Fig. 4B) and DMH (F_(3,12)_ = 2.89; P = 0.0790; Supplementary Fig. 4C).

**Figure 6:**
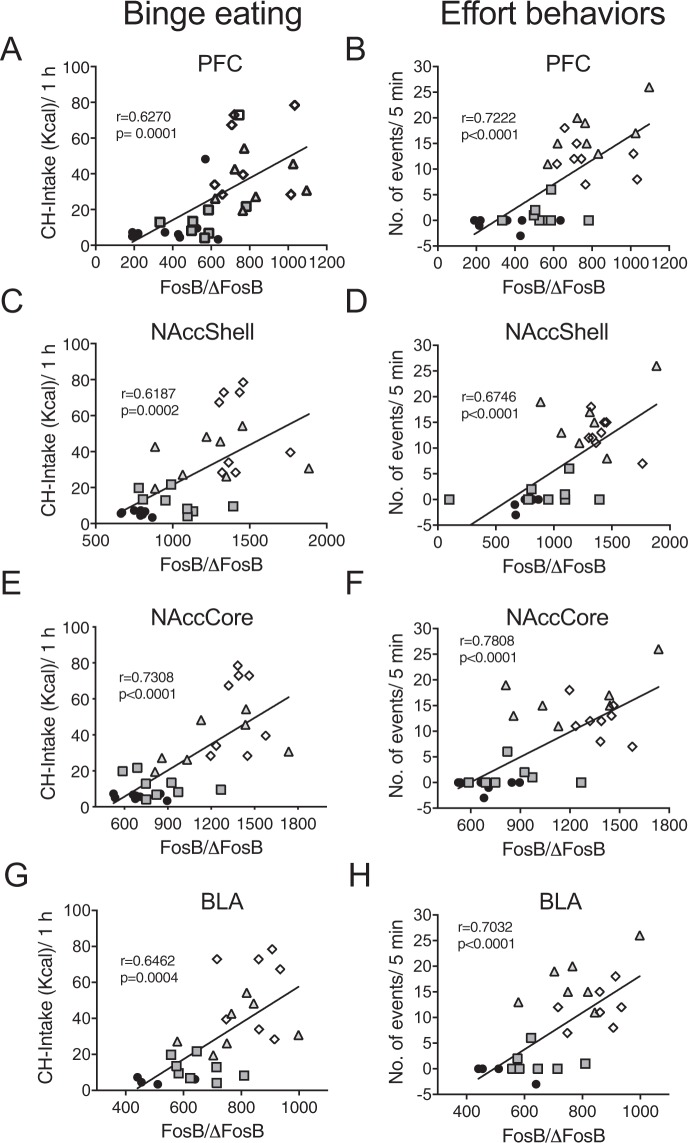
Addiction-like behaviors highly correlated with accumulation of FosB/ΔFosB in corticolimbic areas during week 3 of exposure to PF. Binge eating (left column) significantly correlated with increased FosB/ΔFosB in the four explored areas (**A**) Prefrontal cortex (PFC), (**C**) Accumbens Shell (NAccShell), (**E**) Accumbens Core (NAccCore) and (**G**) Basolateral amygdala (BLA). Likewise, active effort behavior with the wire-mesh box test correlated positively with FosB/ΔFosB expression in all areas (**B,D,F,H**). Black circles = control groups, grey squares = chocolate ad libitum, grey triangles = chocolate random and white diamonds.

At the end of the WDL week no differences were observed between groups in PFC (F_(3,12)_ = 3.151; P = 0.064; Fig. 5A) and BLA (F_(3,12)_ = 0.0436; P = 0.98; Fig. 5D). However, significant differences in FosB/ΔFosB remained between groups in the NAccCore (F_(3,12)_ = 4.984; P<0.017; Fig. 5B) and NAccShell F_(3,12)_ = 4.115 P < 0.03; Fig. 5C). At the end of the 3rd week a significant correlation was found between the intensity of the binge eating behavior and FosB/ΔFosB positive cells in PFC (r = 0.6270 P = 0.0001; Fig. 6A), NAccShell (r = 0.5564; P < 0.0009; Fig. 6C), NAccCore (r = 0.7308; P < 0.0001; Fig. 6E) and BLA (r = 0.6462, P = 0.0004; Fig. 6G). A significant correlation was also found between the total interaction with the wire-mesh box and FosB/ΔFosB positive cells in PFC (r = 0.7222 P < 0.0001; Fig. 6B), NAccShell (r = 0.6746, P < 0.0001; Fig. 6D), NAccCore (r = 0.7808; P < 0.0001; Fig. 6F) and BLA (r = 0.7032, P < 0.0001; Fig. 6H).

## Discussion

Present data confirm that intermittent access to PF in a random or a fixed time schedule induce effort behaviors and binge eating with a similar intensity and both promoted increased FosB/ΔFosB positive cells in corticolimbic brain areas. In both groups the development of addiction-like behaviors to PF was also evidenced by the weekly escalation of PF intake during binge test (Fig. 3C,D). In rats with *ad libitum* access to chocolate (CH-AL), the criteria for binge eating was not achieved during the binge test along the weeks of access and also not during the withdrawal week. Only when the restricted access was paired with a temporal pattern (CH-F), rats were able to develop anticipation in general activity and in core temperature, which indicates a behavioral and physiological readiness for PF ingestion. This agrees with the “limited model” proposed by Corwin *et al*.^2^, which triggered binge episodes.

Rats exposed to intermittent access to chocolate (CH-F and CH-R) developed binge eating behavior, in agreement with previous studies^36,37^. Moreover, escalation was observed along the 3 sessions of the binge test. It is important to note that during the binge test, both restricted groups ingested significantly more chocolate than the control and the CH-AL groups, and both in a similar manner. The same differential effect between an *ad libitum* and an intermittent condition was observed in the effort behavior using the wire-mesh box, confirming that the restricted access, with or without timing, is a key factor to induce effort and motivation behaviors to obtain PF. This in agreement with other studies that described increased motivation and seeking behaviors under restricted access protocols to PF^35,38^. Contrasting, Fiorillo *et al*.^39^, indicate that fixed restricted access to PF may optimize the prediction capacity in the VTA for release of dopamine for the reward response. Here, we did not obtain significant differences in addiction-like responses between CH-R and CH-F conditions.

Anticipatory behaviors were only observed in CH-F rats. Anticipatory activation started 1 h prior and reached peak values at the moment of chocolate access. Contrasting, anticipation was not observed in CH-R rats lacking a predictable scheduled of PF access, not even on day 21 after a scheduled access at 13 h on day 20, confirming that the temporality and regularity of the reward access is required for anticipation and involves a temporal based mechanism^40^. The development of anticipatory activation is considered the behavioral basis for craving because it enables expectation and seeking for the reward stimulus^3^. Chocolate is a palatable food with highly rewarding effects in humans and experimental models and it is strongly associated with craving behavior especially when paired with stressful conditions^41^. The rewarding and craving effects of chocolate are due to psychoactive substances triggered in brain areas involved in the modulation of appetite, reward and mood^42^. Studies using restricted access to regular food^9,27^ or palatable food^16,22^ reported that feeding schedules induce a high activation of c-Fos and clock genes in corticolimbic and hypothalamic brain structures involved in homeostatic and reward control of food intake. This mechanism may underlie a temporal estimation system that synchronizes metabolism and behavior to the reward access and organizes the behavioral outcome in time^10,22^. This food entrained anticipation persists for at least 7 days after interrupting the chocolate access, indicating the continuation of a clock related system that may drive the seeking and expectation behavior for reward^43^. Also, this persisting mechanism may drive the seeking and craving behaviors to reward during the withdrawal period^8,39,44^.

A surprising finding was the anticipatory decrease of body temperature starting as early as 6 h and followed by the progressive increase preceding chocolate access in the CH-F group. This process provides a new insight into the anticipation to PF because it suggests that the anticipatory process to PF starts much earlier than observed with the behavioral activation. Low body temperature has been reported as part of the withdrawal response in rodents that have been exposed to drugs^45,46^. Thus, in the present study this anticipatory low temperature may indicate a daily brief withdrawal process due to the restricted access. However, this low temperature pattern was not observed during the week of chocolate withdrawal, thus, suggesting a process elicited by the short PF intake. This early temperature decrease has been described in food-entrained rodents^12,47^.

Therefore, rats may undergo a voluntary fasting interval several hours before chocolate access in order to be able to binge chocolate at the predicted time. The mechanisms underlying this anticipatory response will require further studies and will provide new insights on the timing system underlying anticipatory activation and craving.

Interestingly the CH-AL group ingested daily large amounts of chocolate along the 24 h cycle and did not exhibit any of the behavioral criteria associated with food addiction- like behaviors to PF, confirming the relevant role of restriction and temporality. This group, however, showed increased general activity during the light phase associated with an increased chocolate intake. Previous studies have described that a high fat diet modifies daily general activity patterns, increasing activation and food intake during the light phase, which can lead to chronodisruption^48,49^

After chocolate ingestion an increase of FosB/ΔFosB was observed in the 4 brain areas explored in this study as previously described for Fos family proteins after acute administration of drugs of abuse^30^. While FosB is an unstable protein, that degrades after a few hours, its alternative splicing leads to ΔFosB expression^31^, which is a stable protein and accumulates in the same cells. The accumulation of ΔFosB is a transcription factor promoting dendritic growth and enhancing synaptic contacts. Such changes are suggested to initiate and maintain neuronal changes for an addicted state^30^. On week 3 positive cells expressed a combination of FosB/ΔFosB according to the antibody used in the present study. Because brains were collected 1 h after chocolate ingestion, high levels of FosB may have been triggered by the acute effect of chocolate intake for CH-R and CH-F groups. This may have combined with the accumulation of ΔFosB after 3 weeks of PF exposure. Structural plastic changes in the reward circuitry are suggested to underlie craving and compulsive ingestion behaviors^28,50,51^. Other regions explored (PVT, DHM and ARC) did not show significant changes of FosB/ΔFosB, this may be due to the time point in which brains were obtained and that does not represent the anticipatory activation. Moreover, an increase of FosB/ΔFosB has not been described with relation to ingestion of PF in these brain regions.

The enhanced expression of FosB/ΔFosB in the corticolimbic areas and the positive correlation with binge eating and effort behaviors for PF suggest a role of this protein complex in the development of addiction-like behaviors.

Moreover, after the withdrawal week, positive cells only expressed accumulation of ΔFosB exclusively and this was only observed in the NAcc Shell and Core in the CH-R group. The accumulation of ΔFosB during withdrawal is suggested to mediate the seeking reward behavior and relapse to drugs^24^. Importantly, ∆FosB increase did not persist in the PFC and BLA after 7 days of chocolate interruption, suggesting the start of an extinction process. Schultz *et al*.^39^, have suggested that when a reward is expected but not presented, the activation of dopaminergic neurons is reduced below the regular intensity. This may explain the reduced level of FosB/ΔFosB observed in the WDL brains.

## Conclusion

We provide evidence that intermittent access to PF in a fixed or random schedule can lead to addiction-like responses and accumulation of FosB/ΔFosB, which is used as biomarker of addiction. Here we confirmed that restricted access to PF is a key factor triggering effort behaviors and binge eating and we provide evidence that adding up a temporal predictable schedule allowed the development of behavioral and physiological anticipation (Table 1). Present findings point out that scheduled activities affecting the reward system may play a relevant role in promoting the formation of habits towards addiction-like behavior. The mechanisms underlying this timing system require further research in order to better understand the factors that contribute to behavioral and physiological adaptations for addictive-like behavior.

**Table 1:** Compulsive eating behaviors to palatable food.

Condition/Addiction like-criteria	Control (CTRL)	Chocolate ad libitum (CH-AL)	Chocolate random (CH-R)	Chocolate fixed (CH-F)
Activity anticipation (craving)	✗	✗	✗	✓ W
Temperature anticipation	✗	✗	✗	✓
Binge eating-like behavior	✗	✗	✓ W	✓ W
Effort/Motivated behaviors	✗	W	✓ W	✓ W
ΔFos increase	✗	✗	✓ W	✓ W

Restricted access to chocolate with and without timing, can reflect the key role of a predictable schedule to develop and maintain addiction like-behaviors through the induction of plastic changes in the reward system. ✗ indicate absence of the criteria, the ✓ indicate presence of the criteria and W indicate the persistence or presence of the criteria after the withdrawal period.

## Materials and Methods

### Animals

Male Wistar rats, weighting 230–260 g at the beginning of the experiment, were obtained from the bioterium in the Faculty of Medicine in the National Autonomous University of Mexico (UNAM). Rats were housed individually in transparent acrylic cages (45 cm × 30 cm 20 cm) with free access to water and chow food (Rodent Laboratory Chow 3.06 Kcal/g) and water; temperature was maintained at 21 ± 1 °C, under a 12:12 h light/dark cycle (light onset at 7:00 h) and continuous air flow. The project was approved by the Research and Ethics Committee of the Medical Faculty, UNAM (number: 157/2014). Procedures agree with international guidelines for the ethical use and handling of experimental animals.

### Experimental design

Before starting the experimental procedures, all rats were acclimated to the housing conditions for 1 week. Experimental animals were randomly assigned to one of the four groups and were maintained in the corresponding condition for 21 days:

1. Control rats (CTRL) had no access to PF.
2. Chocolate *ad libitum* rats (CH-AL) had free unrestricted access to chocolate (Kinder Chocolate Maxi © Ferrero de México, S.A. de C.V. 28.6 Kcal, 10.3% proteins, 54.2% carbohydrates and 35.5% fat), together with chow food along the 24 h.
3. Chocolate Fixed rats (CH-F) received 5 g of chocolate daily at a fixed schedule (13 h).
4. Chocolate Random rats (CH-R) had daily restricted access to 5 g of chocolate that was placed on the feeder according to a random unpredictable schedule (see Table 2); the majority of chocolate events were programmed to the day (one piece/day) in a time window between events ranging from 18 to 36 h. With the aim to compare the anticipatory activation between CH-F and CH-R rats, on day 20 of chocolate access the CH-R group received the 5 g of chocolate at 13 h (as CH-F rats). The following day (day 21) anticipation was compared between both restricted groups.

**Table 2:** Chocolate random access program.

Day/Week	1st	2nd	3rd
MONDAY	20:00 h	13:00 h	09:00 h
TUESDAY	15:00 h	22:00 h	06:00 h
WEDNESDAY	18:00 h	11:00 h	17:00 h
THURSDAY	12:00 h	16:00 h	19:00 h
FRIDAY	10:00 h	07:00 h	08:00 h
SATURDAY	13:00 h	12:00 h	16:00 h
SUNDAY	14:00 h	21:00 h	13:00 h

Another series of rats underwent the same protocol and after 21 days, the chocolate access was interrupted for 7 days in order to evaluate a withdrawal condition (WDL). Criteria to score addiction-like behavior where: anticipatory activation, binge eating and effort behaviors to obtain chocolate. In corticolimbic areas ΔFosB immunoreactive (IR) cells were counted.

### Monitoring of general activity and core temperature

Before starting the baseline, a set of rats (n = 8/group) underwent surgery to introduce an intra-abdominal temperature sensor (iButton sensor-temperature logger; Maxim integrated products, USA). One week before starting baseline, rats were anaesthetized with an intramuscular dose of xylazine (Procin 0.01 ml/100 g) and ketamine (Inoketam 0.02 ml/100 gr). Under deep anesthesia a small incision was performed in the abdominal cavity and the temperature sensor, previously sterilized, was introduced in the peritoneum. Abdominal muscles were sutured with absorbable catgut (000) and skin was sutured with surgical suture (Atramat, International Farmaceutica, SA. de CV. Mexico). Rats were left for 1 week to recover before starting the baseline.

In order to evaluate the daily patterns in general activity and anticipatory activation, rats were housed in individual cages over tilt sensors. The sensors detected continuously activity counts, which were collected with a digitalized system and automatically stored in a computer. Analysis was performed with SPAD9 (Data processing system, 1.1.1 version; Omnialva SA. De CV. Mexico City, Mexico) based on MATLAB. Activity counts are represented as actograms and as activity waves. Due to differential sensitivity of the tilt sensors, data were normalized to the proportional percentage of the daily activity, normalized data were used to elaborate mean daily activity waves and for the anticipatory analysis.

Core temperature was collected every 30 min. At the end of the experiments, rats were perfused and iButtons were extracted. Data were organized by weeks and are represented as mean daily activity waves and heat maps. Heat maps were obtained with the software El Temps (Circadian data processing system developed by Antoni Díez-Noguera.

### Anticipation to palatable food

Anticipatory activation was evaluated during the 3^rd^ week of chocolate access and for the withdrawal week. Daily general activity and temperature events were organized in 60-min intervals and are represented as temporal graphs with data for the 6 h prior to chocolate delivery (7–12 h) and the hour during chocolate intake access (13 h). To better evidence the light phase activation elicited by chocolate access, for weeks 1, 2, 3 and withdrawal the total activity during the 12 h of the light phase were compared with the light phase activity in the BL. This is represented as the delta of change (Δ) with the base line. Also, for day 21 the anticipatory activation and temperature were compared between CH-R and CH-F groups.

### Binge eating test

Another set of rats under similar conditions (n = 8/group) was exposed for 3 weeks to the protocol. At the end of each experimental week, on Fridays, rats were exposed to a complete bar of chocolate (21 g/118 kcal) for one hour (13–14 h). The amount of ingested chocolate was assessed in grams and was transformed to kcal. Along the days also the normal chow ingestion was assessed in order to determine their average caloric intake. Binge eating was considered when rats ingested 20% or more kcal from their daily kcal intake in 1 h. Data are shown as mean kcal in 1 h for the BL, weeks 1, 2, 3 and WDL week.

### Effort-test with a wire-mesh box

The aim of this test was to evaluate the interaction and effort displayed by rats in order to obtain the PF. A different set of rats was used for this test (n = 9/group). Rats were tested at the end of the 3rd week of chocolate exposure and at the end of the WDL week. The wire-mesh box was 5 cm × 5 cm ×5 cm with spaces in the mesh of 5 mm × 5 mm, which allowed the rats to see and smell the chocolate but not to touch or bite it^16^.

On experimental day 20, rats were individually exposed in their home cage to an empty box for 5 min. The following day (day 21) rats were exposed to the same box containing 5 g of chocolate. The box was introduced in the home cage at 12:55 h for 5 min and was removed from the home cage at 13 h. Behavioral events were recorded with a digital camera and evaluated with an instantaneous sampling strategy to measure the approaches and interaction with the box as previously reported^16^. Briefly, the videos were stopped every 5 sec and the behavior performed by the animal was annotated; 60 samples were obtained. Behavior was classified in 2 categories: 1. Passive interaction with the box: smell or contact with the paws; 2. Active-effort interaction: bite, pull/push, manipulation of the cage with forepaws and attempts to open the box. The total interaction resulted from adding passive behaviors + active-effort behaviors. The number of events obtained with the empty box (first test day) was subtracted from the number of events obtained with the box containing chocolate (second test day). Videos were analyzed by two investigators who were blind of the experimental condition. Data are represented as medians and ranks.

### Immunohistochemistry for FosB/ΔFosB

Brains were obtained (n = 6/group) on day 21 of chocolate exposure and another series at the end of the WDL period. All brains were obtained at 14 h (1 h after chocolate access).

Rats were anaesthetized with an overdose of pentobarbital (Pinsabental; 1.5 ml/300 gr.) and perfused intracardially with 250 ml saline solution (0.9%) followed by 250 ml of paraformaldehyde in 4% in 1 mM buffer phosphate (PB; pH 7.2). Brains were extracted and postfixed for 1 week and cryoprotected in 30% of sucrose solution for another week. The brains were sectioned in 40 µm with a cryostat and collected in 4 series. One series was incubated in rabbit polyclonal ∆FosB/FosB primary antibody (Santa Cruz Biotechnology, Dallas TX, USA) diluted 1:1500 in PB saline 0.9%, 0.25 g nutritive gelatin and 0.5% Triton X-100 (PBSGT) for 72 h. This was followed by incubation with secondary antibody (IgG) anti-rabbit (Vector Laboratories CA, USA) diluted 5:1000 µl in PBSGT for 90 min, then incubation for 90 min in avidin-biotin- peroxidase complex (9:1000) (AB; vector Laboratories Inc.) and were reacted in a dilution of chromogen diaminobenzidine DAB (5 mg/1 ml) in Trizma pH 7.2 and 30% peroxide (H_2_ O_2,_ 35 µl) during 10 min. Between incubations sections were rinsed 3 times in PBS. Sections were mounted on gelatin-coated slides, were dehydrated through a series of alcohols cleared with xylene and covered with Entellan (Merk).

The number of ∆FosB positive cells were counted in the prefrontal prelimbic region (PFC; 3.5 mm, and 2.5 mm anterior to Bregma), in the nucleus accumbens core (NaccCore) and nucleus accumbens shell (NaccShell; 1.89, 1.6 and 1.2 mm anterior to Bregma) and in the basolateral amygdala (BLA; −1.56, −1.92, −2.28 mm from Bregma) according to the stereotaxic atlas^52^. In order to verify a possible effect on hypothalamic nuclei involved in metabolic and temperature regulation and the hypothalamic-corticolimbic interaction, additional sections were analyzed containing the ARC, DMH and PVT (−2.92, −3.00, −3.12 mm from Bregma). Sections were identified with an optical microscope (Leica ICC50 HD), photographs were acquired with a 20x magnification using a camera and software LAS EZ (Leica Application Suite). Immunopositive ∆FosB positive cells were counted with the Image J Launcher (NIH) program (MedianCybernetics, INC.).

### Statistical analysis

General activity and temperature are represented as mean temporal patterns for the 6 h preceding chocolate access. Anticipatory changes for 1 h intervals were compared with a two-way ANOVA, to compare among groups with hours as a variable of repeated measures. Delta change analysis in the light phase for BL, 1, 2, 3 and WDL week were compared with a one-way ANOVA for repeated measures (RM). To analyze the last day of chocolate exposure for CH-R and CH-F groups were compared in 1 h intervals with two-way ANOVA with hours as variable of RM. All ANOVAs were followed by Tukey post hoc test with α < 0.05.

For the binge eating test data are represented as mean ± standard error of the mean (SEM) and values for the weeks were compared with a one-way ANOVA for RM followed by a Tukey post hoc with α < 0.05. Wire-mesh box test data are represented as median with ± ranks and groups were compared with a Kruskal Wallis test by ranks, followed by Dunn post hoc test with P < 0.05. Cell count of FosB/ΔFosB was arranged per region and group and are represented as mean ± SEM. Groups were compared with a one-way ANOVA followed by a Tukey post hoc test with α < 0.05. To assess association between addiction-like behaviors (binge eating and effort/motivation behaviors) and FosB/ΔFosB immunopositive cells in the different corticolimbic areas a Pearson correlation coefficient was performed. All statistical analyses were performed with the Graph Prism 6 software, Windows Version.

## Supplementary information

Dataset 1

## Data Availability

All data generated and analyzed as a part of this study are included within this article (and its supplementary information files).
