# Supplementary material for: Random access to palatable food stimulates similar addiction-like responses as a fixed schedule, but only a fixed schedule elicits anticipatory activation
Source: Sci Rep. 2019 Dec 3;9:18223. doi: 10.1038/s41598-019-54540-0 (PMC6890727; doi:10.1038/s41598-019-54540-0)
Supplement: Supplementary file 1 — Dataset 1 [file 41598_2019_54540_MOESM1_ESM.docx]

Random access to palatable food stimulates similar addiction-like responses as a fixed schedule, but only a fixed schedule elicits anticipatory activation

Geovanni Muñoz-Escobar, Natalí N Guerrero-Vargas, Carolina Escobar*

Departamento de Anatomía, Facultad de Medicina, Universidad Nacional Autónoma de México, Ciudad de México, México

***Corresponding Author:**

Carolina Escobar

Departamento de Anatomía

Facultad de Medicina UNAM

Av Universidad 3000

Ciudad Universitaria

CDMX 04510

Fax number: 5623 2422

Telephone number: 5623 0222 ext 45062

e-mail address: [escocarolina@gmail.com](mailto:escocarolina@gmail.com)

**FIGURE S1**

**Supplementary Figure 1.** Representative double-plotted actograms and weekly average general activity profiles during base line (BL) and 3 weeks of chocolate exposure. (A)Representative actogram and (B) activity profile for control rats (CTRL). (C) Chocolate-*ad libitum* group (CH-AL) representative actogram and (D) activity profiles, the red zone represents the interval when chocolate was placed on the feeder. (E) Representative actogram and (F) activity profiles of Chocolate random access rats (CH-R), the red triangles indicate the time when chocolate was placed in the feeder. (G) Chocolate fixed rats (CH-F) representative actogram and (H) activity profiles, the red line indicates the daily schedule of chocolate delivery (13:00 h). White and dark horizontal bars represent the light-dark cycle.

**FIGURE S2**

**Supplementary Figure 2.** Representative double-plotted core temperature heat maps and weekly average profiles during base line (BL) and 3 weeks of chocolate exposure.

All groups showed of a core temperature rat under light-dark cycle with chow and water *ad libitum*. (A) Representative heat map and (B) 24h temperature profile for control rats (CTRL). (C) Chocolate-*ad libitum* group (CH-AL) representative heat map and (D) 24h temperature profile, the red rectangle represents chocolate access. (E) Representative heat map and (F) 24h temperature profiles of Chocolate random access rats (CH-R), the black rectangles indicate chocolate delivery. (G) Chocolate fixed rats (CH-F) representative heat map and (H) 24h temperature profiles, the black line indicates the daily schedule of chocolate delivery (13:00 h). White and dark horizontal bars represent the light-dark cycle.

**FIGURE S3**

**Supplementary Figure 3**. (A) Body weight from baseline (BL), during the three weeks of chocolate access and the withdrawal week (WDL) for control (CTRL), chocolate-*ad libitum* group (CH-AL), chocolate random access rats (CH-R) and chocolate fixed access rats (CH-F). Data are expressed as mean ± SEM (n=8/group). The Tuckey post hoc test indicated statistical difference (P<0.05) of CH-R vs CTRL indicated with #, CH-F vs CTRL indicated with +. (B) Daily kilocaloric consumption of chow (black bars) and chocolate (dotted bars) during the BL, three weeks of chocolate exposure and WDL phase. CH-R and CH-F groups received daily a piece of 5g of chocolate in the middle of the light phase, during the three weeks of the chocolate exposure phase.

**FIGURE S4**

**Supplementary Figure 4.** Number of FosB/ΔFosB positive cells at the end of the 3^rd^ week of chocolate exposure in the A) Paraventricular thalamus, B) Arcuate nucleus and C) Dorsomediual hypothalamus. Control (CTRL), chocolate ad-libitum (CH-AL), chocolate random (CH-R) and chocolate fixed (CH-F) rats. Data are expressed as mean ± SEM (n=4-5/group).
